# Mechanisms by which tryptophan metabolites enhance intestinal barrier function to prevent necrotizing enterocolitis in preterm infants

**DOI:** 10.3389/fimmu.2026.1782006

**Published:** 2026-04-13

**Authors:** Xinxin Cui, Jianli Qiu, Yan Xu, Zhiwei Guan, Ying Ding, Suping Yu, Shuhua Fan, Guihua Song, Xianqing Ren

**Affiliations:** 1 Pediatric Hospital, The First Affiliated Hospital of Henan University of Chinese Medicine, Zhengzhou, China; 2 Pediatric Medical College, The First Affiliated Hospital of Henan University of Chinese Medicine, Zhengzhou, China

**Keywords:** Aryl hydrocarbon receptor (AhR), indole metabolites, Necrotizing enterocolitis (NEC), tryptophan metabolism, intestinal barrier

## Abstract

Necrotizing enterocolitis (NEC) is one of the most severe intestinal diseases affecting preterm infants, characterized by high mortality rates and significant long-term complications, which present substantial challenges to clinical management. The primary pathological mechanism underlying NEC is the immature development of the intestinal barrier in preterm infants, which fails to adequately resist luminal pathogen invasion, triggering uncontrolled inflammatory responses and tissue damage. Additionally, intestinal dysbiosis further compromises the integrity of the intestinal barrier. In recent years, growing attention has been given to the role of tryptophan metabolites in maintaining intestinal barrier integrity. As an essential amino acid, tryptophan metabolites—particularly those derived from gut microbiota, such as indole compounds—have been shown to exert significant barrier-protective effects through multiple mechanisms, including the activation of the aryl hydrocarbon receptor (AhR), upregulation of tight junction protein expression, inhibition of inflammatory responses, and promotion of epithelial cell repair. This review aims to summarize the major biological pathways of tryptophan metabolism, with particular emphasis on the molecular mechanisms by which microbial indole derivatives enhance the physical, chemical, and immune barriers. By synthesizing both preclinical studies and clinical evidence, this article explores the translational potential of tryptophan metabolite-targeted strategies for NEC prevention, providing a theoretical foundation for the development of precise preventive interventions.

## 1 Introduction

Necrotizing enterocolitis (NEC) is a common and severe intestinal inflammatory disease that primarily affects preterm infants, particularly those with very low birth weight (VLBW) (1). Its pathological features include intestinal mucosal necrosis, excessive activation of inflammatory responses, and severe impairment of intestinal barrier function (1, 2). Despite significant advances in perinatal medicine and neonatal intensive care technology, NEC incidence and mortality rates remain unacceptably high. Epidemiological data indicate that NEC affects approximately 5%-22% of very low birth weight infants (3), with an overall mortality rate of 23.5% (4). Infants requiring surgical intervention have particularly poor prognoses, with mortality rates reaching 50.9%, and survivors often experience severe long-term complications, including neurodevelopmental delay and short bowel syndrome (3, 4).

Traditional perspectives have viewed NEC as an infection-triggered excessive immune response. However, emerging pathological insights indicate that the core of NEC pathogenesis is not merely uncontrolled inflammation, but rather a vicious cycle driven by the structural failure of the immature intestinal barrier, compounded by disruption of the local metabolic microenvironment (5, 6). The preterm infant intestine exists in a unique hypoxic-ischemic microenvironment where mitochondrial oxidative phosphorylation in intestinal epithelial cells is impaired, severely restricting ATP synthesis (7). This cellular energy crisis prevents immature epithelial cells from maintaining high-energy cytoskeletal rearrangement processes, directly inducing endocytosis and degradation of key tight junction proteins such as Occludin and ZO-1, leading to physical barrier collapse (8, 9). Concurrently, characteristic gut dysbiosis in preterm infants (delayed colonization by beneficial bacteria such as Bifidobacterium and reduced microbial diversity) further exacerbates this process (10). The collapse of the microbiome not only increases susceptibility to pathogens but also causes a deficiency in critical protective metabolites, blocking key signals required for epithelial cell repair, thereby depriving the intestine of the metabolic flexibility needed to maintain homeostasis under dual insults of hypoxia and metabolic dysregulation (11, 12).

Among potential intervention targets for reshaping the intestinal metabolic microenvironment, tryptophan (Trp) and its microbiota-derived metabolites have attracted considerable attention due to their significant barrier repair and immunomodulatory capabilities. As key chemical messengers mediating host-microbiota interactions, indole derivatives produced via microbial tryptophan metabolism—such as indole-3-propionic acid (IPA) and indole-3-lactic acid (ILA)—serve as high-affinity endogenous ligands for the host aryl hydrocarbon receptor (AhR) (13–15). Existing studies demonstrate that these metabolites can reinforce the intestinal barrier through multiple dimensions by activating AhR signaling pathways:At the molecular level, they upregulate the expression of tight junction proteins (ZO-1, Occludin) and downregulate the pore-forming protein Claudin-2; at the cellular level, they enhance mitochondrial metabolism and suppress the NF-κB-mediated inflammatory cascade (8, 15, 16). Notably, human milk, the “gold standard” for NEC prevention, exerts its protective effects largely through the active remodeling of the neonatal intestinal metabolome by naturally occurring AhR ligands (17, 18).

Although adult studies have fully established the critical regulatory role of tryptophan metabolic homeostasis on intestinal barrier function (19), and animal studies clearly demonstrate that tryptophan metabolites abundant in human milk can effectively prevent NEC in mouse models (17), the specific mechanisms by which this metabolic axis contributes to NEC prevention in preterm infants have not been systematically elucidated. The unique intestinal microenvironment of preterm infants (including hypoxia, inflammation, and dysbiosis) may significantly affect the bioavailability and mechanisms of action of tryptophan metabolites. Therefore, systematically elucidating the molecular mechanisms by which tryptophan metabolites reinforce the immature intestinal barrier holds important theoretical and clinical value for developing precise NEC prevention strategies based on tryptophan metabolites.

Accordingly, this review aims to: (1) systematically outline the major pathways of tryptophan metabolism and their biological functions; (2) deeply analyze the molecular mechanisms by which tryptophan metabolites reinforce the intestinal barrier, including regulation of key signaling pathways and transcription factors; and (3) integrate existing clinical and basic research evidence to evaluate the feasibility and clinical translation prospects of tryptophan metabolites as NEC prevention strategies.

## 2 Physiological characteristics and barrier vulnerability of intestinal development in preterm infants

Immature intestinal development in preterm infants represents the fundamental predisposing factor for necrotizing enterocolitis (NEC). Compared to term infants and adults, preterm infants exhibit significant deficiencies in three dimensions: the physical barrier, the immune barrier, and the microbial ecosystem. These three aspects are interwoven, collectively constituting the pathophysiological basis of intestinal barrier vulnerability in preterm infants.

### 2.1 Physical barrier: structural defects and energy metabolic impairment

The fragility of the physical intestinal barrier in preterm infants stems from dual defects: developmental structural immaturity and impaired energy metabolism. Late gestation (24–40 weeks) represents a critical window for rapid intestinal epithelial maturation, including villus morphogenesis, microvillus differentiation, and tight junction complex assembly. Preterm infants are prematurely separated from the maternal environment, interrupting this developmental process and resulting in incomplete epithelial structure with significantly lower expression of key tight junction proteins such as Occludin and ZO-1 compared to term infants, leading to loose intercellular connections and increased permeability. More critically, preterm infant intestinal epithelial cells universally exhibit mitochondrial dysfunction and impaired ATP synthesis (7). Tight junction assembly, maintenance, and cytoskeletal rearrangement are highly energy-intensive processes; this energy crisis can directly induce the upregulation of Cldn-2 and the endocytosis and degradation of Occludin and ZO-1, ultimately resulting in physical barrier collapse. Additionally, preterm infants possess insufficient intestinal stem cell reserves and exhibit low proliferative and differentiative activity, resulting in repair capacity after epithelial injury that is far weaker than in adults; once the barrier is disrupted, self-reconstruction is difficult to achieve (20).

### 2.2 Immune barrier: tolerance defects and pro-inflammatory bias

The preterm infant intestinal immune system exists in a “naïve” state, with core defects in immature immune tolerance mechanisms and underdeveloped inflammatory regulatory networks. Unlike the adult intestine, which has established robust commensal tolerance, the preterm infant intestine has fewer regulatory T cells (Tregs) with impaired function and insufficient secretion of the anti-inflammatory cytokine IL-10. Concurrently, recognition sensitivity to pathogen-associated molecular patterns (PAMPs) is abnormally elevated, leading to excessive release of pro-inflammatory factors such as TNF-α, IL-6, and IL-8 (20, 21). This “pro-inflammatory bias” causes the preterm infant intestine to be highly susceptible to uncontrolled inflammatory cascades when stimulated by microbiota —even by normal commensal bacteria—which directly damages the epithelium and disrupts the barrier. Notably, the first three months after birth represent a critical period for “education” of the intestinal immune system through microbial stimulation, but early dysbiosis in preterm infants severely hinders this developmental process (22).

### 2.3 Microbial barrier: delayed colonization and metabolic functional deficiency

The pattern of intestinal microbiome establishment in preterm infants differs fundamentally from that in term infants. Influenced by multiple factors, including cesarean delivery, antibiotic exposure, delayed enteral nutrition, and the closed NICU environment, the preterm infant intestine exhibits characteristic dysbiosis featuring “delayed colonization by beneficial bacteria such as Bifidobacterium, overgrowth of potential pathogens such as Proteobacteria, and low overall microbial diversity” (23, 24). However, the consequences of this dysbiosis extend far beyond increased risk of pathogen invasion. The deeper pathological significance lies in the fact that the absence of beneficial bacteria directly leads to microbial metabolic functional deficiency—severely insufficient production of protective metabolites such as short-chain fatty acids and indole derivatives (11). These metabolites are essential signaling molecules required for host epithelial repair, tight junction strengthening, and immune tolerance maintenance. The absence of these metabolic signals deprives the preterm infant intestine of the “metabolic flexibility” necessary to maintain barrier homeostasis under the dual insults of hypoxia and infection (12).

In summary, physical barrier defects, immune tolerance bias, and microbial metabolic functional deficiency are mutually causal, collectively constructing the pathophysiological basis of NEC in preterm infants. This systemic vulnerability suggests that anti-infective therapy alone is unlikely to be effective, and that restoring metabolic-immune homeostasis in the intestine—particularly reinstating the protective effects of tryptophan metabolites—may represent a critical breakthrough in the prevention of NEC.

## 3 Tryptophan metabolic pathways and their biological functions

Tryptophan is one of the essential amino acids in the human body that cannot be synthesized endogenously and must be obtained through dietary intake. Tryptophan is metabolized *in vivo* primarily through three pathways: the host-mediated kynurenine pathway, the serotonin pathway, and the gut microbiota-driven indole pathway. Metabolites produced by these three pathways play important roles in regulating immune responses, oxidative stress, intestinal barrier function, and neurotransmitter synthesis. The balance of tryptophan metabolism is crucial for maintaining organismal homeostasis, and dysregulation of any pathway can lead to disease development.

### 3.1 Kynurenine metabolic pathway: immunoregulatory and neurotoxic effects

The kynurenine metabolic pathway represents the major route of tryptophan metabolism, with approximately 95% of tryptophan being metabolized through this pathway. Under inflammatory or stress conditions, the rate-limiting enzyme indoleamine 2,3-dioxygenase 1 (IDO1) is strongly induced by cytokines such as interferon-γ, converting tryptophan to N-formylkynurenine, which is further processed to generate downstream metabolites including kynurenic acid and quinolinic acid (25). This process has dual immunological effects: on the one hand, it induces T cell starvation-mediated apoptosis through local tryptophan depletion; on the other hand, its downstream products (including kynurenic acid) can serve as AhR ligands to induce Treg differentiation, collectively mediating immune tolerance mechanisms (25, 26). However, excessive activation of this pathway also generates neurotoxic metabolites, such as quinolinic acid, which is closely associated with neurodegeneration (27). Additionally, the liver-specific enzyme tryptophan 2,3-dioxygenase (TDO2), regulated by glucocorticoids and cytokines, participates in maintaining systemic tryptophan homeostasis (28). Notably, recent studies have found that the kynurenine pathway metabolite 3-hydroxyanthranilic acid can alleviate hyperoxia-induced bronchopulmonary dysplasia by inhibiting ferroptosis (29), suggesting that metabolites from this pathway have potential value in multi-organ protection in preterm infants.In summary, the kynurenine pathway, regulated by the rate-limiting enzymes IDO1 and TDO2, maintains a dynamic balance between immune tolerance and neurotoxicity, and the diversity of its metabolites underscores its broad translational potential in multi-organ protection and disease intervention.

### 3.2 Serotonin pathway: bidirectional regulation of intestinal motility and secretory function

The serotonin (5-hydroxytryptamine, 5-HT) pathway accounts for approximately 5% of tryptophan metabolism and is initiated by tryptophan hydroxylase (TPH), which converts tryptophan to 5-hydroxytryptophan, followed by catalysis by aromatic L-amino acid decarboxylase (AADC) to generate serotonin (30). TPH1, primarily expressed in enterochromaffin cells, is responsible for synthesizing over 95% of peripheral serotonin, while TPH2 is restricted to the central nervous system (31, 32). Studies indicate that physiological concentrations of 5-HT promote goblet cell secretion of mucin MUC2 by activating 5-HT4 receptors, thereby enhancing the chemical barrier while simultaneously regulating intestinal peristalsis to maintain normal intestinal motility (33). However, under hypoxic or inflammatory stress conditions in preterm infants, 5-HT synthesis increases compensatorily while presynaptic serotonin transporter (SERT) function is impaired, leading to abnormally elevated 5-HT concentrations in the local microenvironment. Excessive 5-HT can trigger the NF-κB pathway by activating HTR7 receptors, inducing pro-inflammatory factor release and disrupting epithelial tight junctions, thereby exacerbating intestinal barrier permeability (34). This dose-dependent bidirectional effect suggests that maintaining homeostasis of the serotonin pathway is essential for the proper establishment of intestinal function in preterm infants; therefore, restoring the balance of this pathway by targeting TPH1 expression or 5-HT receptor activity may represent a novel strategy for protecting the intestinal barrier and treating associated diseases in this population.

### 3.3 Microbial indole pathway: core signaling axis for intestinal barrier protection

The microbial indole pathway represents the core route by which gut microbiota metabolize tryptophan to generate indole and its derivatives. Unlike the host-dominated kynurenine or serotonin pathways, this pathway completely depends on the enzymatic network of gut microbiota, converting unabsorbed tryptophan from the small intestine into functionally diverse indole derivatives, including indole-3-propionic acid (IPA), indole-3-lactic acid (ILA), indole-3-acetic acid (IAA), and indole-3-aldehyde (IAld) (35). Studies demonstrate that these metabolites serve as high-affinity endogenous ligands for the host aryl hydrocarbon receptor (AhR), constituting the core molecular hub connecting gut microbiota to host barrier immunity. By activating AhR signaling networks, they achieve multiple biological effects, including barrier repair, immunomodulation, and antioxidant stress responses (36, 37).

It should be noted that the generation of these indole metabolites is not accomplished independently by a single bacterial species but rather results from synergistic metabolism by specific microbial consortia (14). Delayed colonization or absence of these core bacterial populations in the extremely preterm infant intestine directly leads to a deficiency of protective signals. Specifically, IPA-producing bacteria are primarily strict anaerobic spore-formers (such as Clostridium spp. and certain Peptostreptococcus spp.), and due to disrupted ecological succession in the extremely preterm infant intestine, their stable colonization process is often delayed (38). ILA-producing bacteria are dominated by Lactobacillus spp. and infant-type Bifidobacterium, among which Bifidobacterium represents a key beneficial bacterium that is easily “missing” in the preterm infant intestine. Human milk feeding exerts protective effects against necrotizing enterocolitis (NEC) by selectively promoting proliferation of such bacteria through human milk oligosaccharides (39, 40). Additionally, IAA- and IAld-producing bacteria primarily include Bacteroides spp., Clostridium spp., and Lactobacillus reuteri (13, 41). These key bacterial populations (especially Bacteroides) are highly susceptible to significant suppression by broad-spectrum antibiotics in the neonatal intensive care unit (NICU) environment (42).

The aforementioned indole derivatives, which are synergistically produced by specific gut microbiota, exhibit distinct biological effects despite sharing the common property of acting as AhR agonists, each playing a unique role in maintaining intestinal homeostasis through different molecular mechanisms.

#### 3.3.1 Indole-3-propionic acid: barrier enhancement and immunometabolic remodeling

Indole-3-propionic acid (IPA) possesses potent antioxidant and anti-inflammatory activities. At the level of barrier function, IPA reduces intestinal epithelial paracellular permeability by activating AhR, which upregulates the expression of tight junction proteins occludin and ZO-1, while simultaneously downregulating the expression of the pore-forming protein claudin-2 (43). At the immunometabolic level, IPA can drive metabolic remodeling in CD4^+^T cells toward mitochondrial oxidative phosphorylation, enhancing regulatory T cell adaptability and anti-inflammatory function, thereby maintaining intestinal mucosal immune homeostasis (44). Furthermore, studies indicate that maternal supplementation with IPA during pregnancy can alleviate colitis susceptibility in offspring of maternal immune activation models, suggesting that this metabolite has transplacental protective effects and potential value for preventing early neonatal intestinal inflammation (45).

#### 3.3.2 Indole-3-lactic acid: multi-pathway synergistic protection of intestinal barrier

The barrier protection mechanism of indole-3-lactic acid (ILA) involves synergistic regulation of multiple signaling pathways. On the one hand, ILA can downregulate pore-forming protein Claudin-2 expression through the AhR/Nrf2/STAT3 signaling axis, enhancing epithelial barrier tightness and preventing bacterial translocation (8). On the other hand, ILA can activate AhR signaling to reinforce intestinal mucosal immune defense and alleviate age-related inflammatory responses (inflamm-aging), highlighting its core role in maintaining intestinal health throughout the lifespan (46).

#### 3.3.3 Indole-3-acetic acid: regulation of intestinal barrier and systemic metabolic homeostasis

Indole-3-acetic acid (IAA) plays an important role in maintaining intestinal epithelial function and systemic metabolic homeostasis. At the local level, IAA effectively alleviates epithelial barrier dysfunction caused by inflammation or stress by activating AhR signaling pathways, protecting the integrity of intestinal morphological structure (47). At the systemic level, IAA-producing commensal bacteria (such as Clostridium scindens) and their metabolites can reprogram systemic metabolic homeostasis, alleviating inflammatory bowel disease-associated cachexia independent of food intake, with mechanisms involving white adipose tissue browning and inhibition of skeletal muscle protein degradation (48). For preterm infants with NEC complicated by postnatal growth retardation, IAA offers dual potential benefits of barrier repair and metabolic improvement, making it a complementary target for combination intervention strategies.

#### 3.3.4 Indole-3-aldehyde: regulation of immune responses and macrophage polarization

Unlike other indole metabolites whose core mechanism is barrier protection, the primary biological function of IAld lies in regulating innate immune cell plasticity, particularly playing a key role in remodeling inflammatory microenvironments. In ulcerative colitis models, IAld has been demonstrated to inhibit macrophage polarization toward the pro-inflammatory M1 phenotype, thereby blocking amplification of inflammatory cascades (49). By modulating macrophage functional status, IAld helps establish an immune-tolerant environment in the intestinal mucosa, preventing excessive inflammatory responses against commensal microbiota. This mechanism is particularly important for the preterm infant intestine, which has not yet established robust immune tolerance.

### 3.4 Specificity and clinical significance of tryptophan metabolism in preterm infants

**Figure 1:**
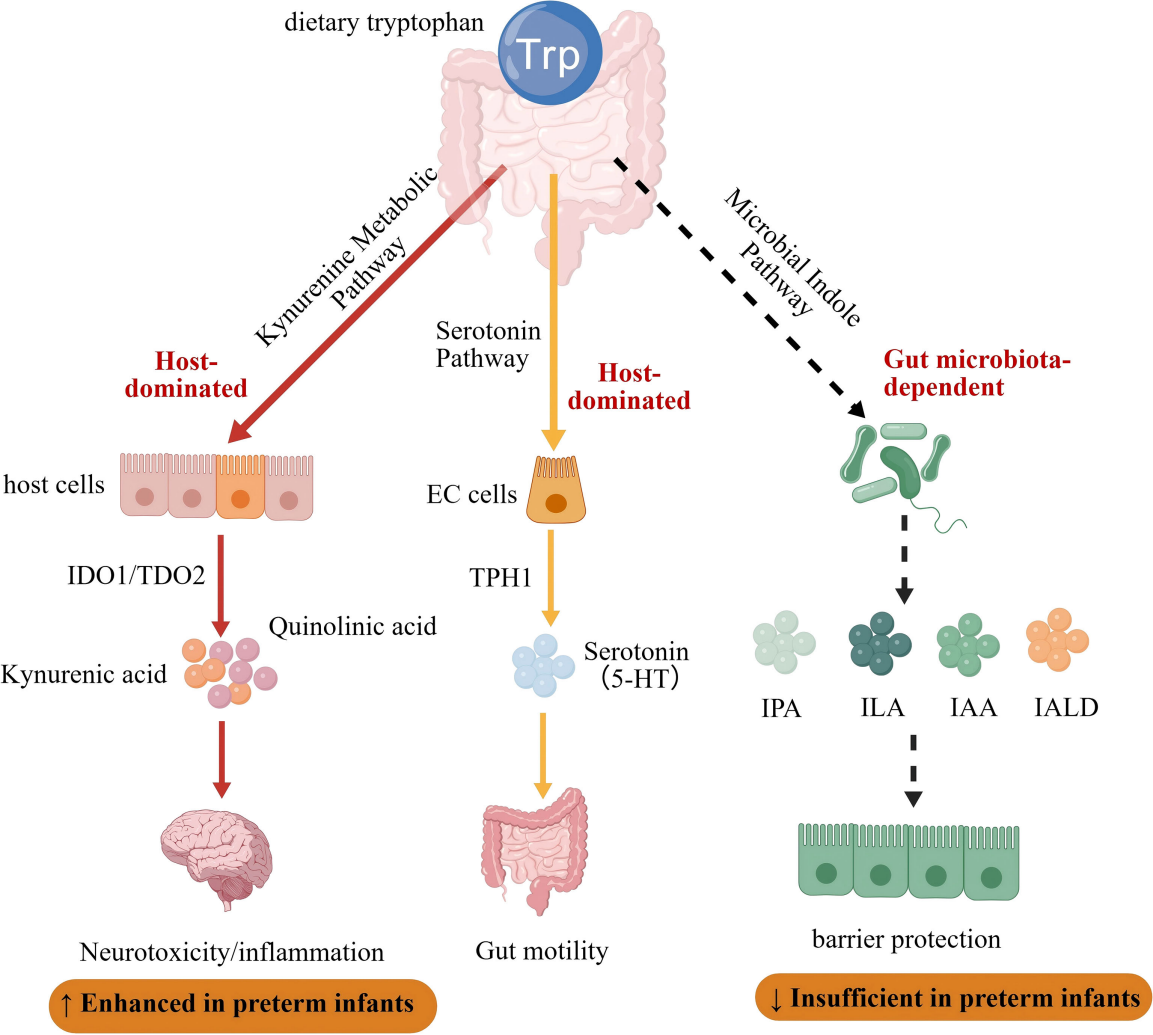
Overview of dietary tryptophan metabolism and its implications in preterm infants. Dietary tryptophan (Trp) is metabolized via host-dominated pathways (serotonin and kynurenine pathways) and the gut microbiota-dependent indole pathway. The serotonin pathway, initiated by TPH1 in enterochromaffin cells, produces 5-HT and regulates gut motility. The kynurenine pathway, regulated by IDO1/TDO2, generates metabolites such as quinolinic acid and kynurenic acid, which are associated with neurotoxicity and inflammation. In preterm infants, host-dominated pathways are enhanced, while the microbiota-dependent pathway is insufficient, leading to reduced barrier protection. Created with BioGDP.com (50).

The core characteristic of tryptophan metabolism in preterm infants lies in functional imbalance among the three major metabolic pathways. The kynurenine and serotonin pathways are dominated by host cells, while the indole pathway completely depends on the enzymatic functions of gut microbiota. For preterm infants, the functional integrity of the indole pathway is directly constrained by gut microbiome maturity, and this pathway represents the sole source of endogenous AhR ligands. The maturation windows of these three pathways in preterm infants differ fundamentally: the kynurenine pathway possesses basal enzymatic activity at birth, whereas the functional integrity of the indole pathway completely depends on the gut microbiota colonization process, with significantly delayed maturation(**Figure 1**). Longitudinal cohort studies reveal that fecal tryptophan metabolite profiles in extremely preterm infants exhibit dynamic evolutionary characteristics, with relative abundances of metabolic pathways significantly influenced by delivery mode and feeding type (12). Compared to term infants, preterm infants exhibit metabolic pathway bias in tryptophan metabolism: kynurenine pathway enzyme activity is relatively enhanced, while microbial indole pathway metabolic flux is significantly insufficient.This metabolic pathway imbalance leads to reduced generation of protective indole metabolites, with AhR signal activation intensity in intestinal epithelium and immune cells falling below physiological requirement thresholds. Clinical evidence demonstrates that overall concentrations of tryptophan metabolites in term infant feces are significantly higher than in preterm infants; specifically, the AhR ligand metabolite indole-3-lactic acid (ILA) is present at significantly higher levels in term infant feces compared to preterm infants (17). Concurrently, immature development of the serotonin pathway in preterm infants further affects normal establishment of intestinal motility and secretory function, resulting in synergistic defects across all three pathways.

This synergistic defect and metabolic bias across the three pathways cause the preterm infant intestine to lack sufficient metabolic buffering capacity to maintain barrier homeostasis when facing stressors such as hypoxia and infection. Therefore, precise intervention targeting the specificity of tryptophan metabolism in preterm infants may emerge as a novel strategy for NEC prevention.

## 4 Molecular mechanisms by which tryptophan metabolites reinforce the intestinal barrier

Tryptophan metabolites reinforce the intestinal barrier through multiple synergistic signaling pathways, forming a multi-level protective system encompassing physical, chemical, and immune barriers. These metabolites—particularly gut microbiota-derived indole compounds—comprehensively enhance intestinal barrier function through mechanisms including activation of the aryl hydrocarbon receptor, regulation of tight junction protein expression, promotion of antimicrobial peptide secretion, and inhibition of inflammatory responses.

Deeply elucidating these molecular mechanisms is of great significance for understanding the role of tryptophan metabolites in preventing necrotizing enterocolitis in preterm infants. This chapter will elaborate on the protective mechanisms of tryptophan metabolites from three levels: physical barrier, chemical barrier, and immune barrier.

### 4.1 AhR signaling axis: core hub for tryptophan metabolite barrier protection

AhR is a ligand-activated transcription factor belonging to the basic helix-loop-helix-Per-ARNT-Sim family, widely expressed in intestinal epithelial cells, immune cells, and vascular endothelial cells. In the unactivated state, AhR binds to chaperone protein complexes, including heat shock protein 90 (HSP90) in the cytoplasm. When indole metabolites produced by gut microbiota serve as high-affinity ligands binding to AhR, AhR undergoes conformational change and translocates to the nucleus. In the nucleus, AhR forms a heterodimer with the aryl hydrocarbon receptor nuclear translocator (ARNT), specifically binding to dioxin response elements (DRE/XRE) on DNA, thereby regulating transcription of barrier-protective factors including cytochrome P450 enzymes, cell cycle regulators, immunomodulatory factors, and barrier function-related proteins (36). Thus, AhR constitutes the core molecular hub connecting gut microbiota tryptophan metabolism to host barrier function.

Notably, the AhR signaling pathway exhibits ligand-dependent effect diversity. Studies indicate that although environmental pollutants such as dioxin isomers are also AhR ligands, they disrupt intestinal epithelial barrier integrity through persistent supraphysiological activation and interference with downstream signal timing. In contrast, microbiota-derived indole metabolites as physiologically occurring endogenous ligands in the gut exhibit reversible, transient, and moderate-intensity binding to AhR, with induced target gene expression patterns coordinated with the body’s own rhythms (51). This fundamental difference in activation patterns reveals the dual regulatory role of AhR on the intestinal barrier: its effect direction is not determined by the receptor itself but rather depends on the chemical nature and signal kinetic characteristics of the ligand. For preterm infants, due to an immature gut microbiome, the integrity of this signaling axis primarily depends on colonization quality and metabolic activity of specific functional bacterial populations (6). This pathological background makes exogenous supplementation of indole metabolites to bypass microbial deficiencies and restore physiological AhR activation intensity an intervention strategy aligned with signaling pathway logic.

### 4.2 Physical barrier: transcriptional regulation and cytoprotection of tight junction proteins

NEC pathogenesis typically begins with abnormal increase in intestinal permeability, known as the “leaky gut” phenomenon. Studies demonstrate that preterm infant intestinal epithelial structure is developmentally immature, and tight junctions are easily disrupted under stimulation from hypoxia, osmotic stress, or pathogenic bacterial colonization, leading to luminal contents and bacterial translocation into the submucosa, triggering destructive inflammatory cascades (52, 53). Tight junctions are the primary structures connecting intestinal epithelial cells, composed of occludin, claudins, claudin family proteins, and junctional adhesion molecules. The expression levels and distribution status of these proteins directly determine intestinal barrier permeability. Claudin 2 is a key protein forming pore-type tight junctions, and its increased expression significantly elevates intestinal permeability.

Studies indicate that tryptophan metabolites reinforce the intestinal physical barrier through dual synergistic mechanisms. Research has confirmed that indole-3-propionic acid (IPA) can upregulate Occludin and ZO-1 expression while downregulating pore-forming protein Claudin-2 by activating AhR, thereby reducing intestinal epithelial paracellular permeability (54, 55). Notably, the protective effect of IPA in mild colitis models primarily depends on barrier enhancement rather than direct anti-inflammatory action (54), indicating that AhR activation directly drives physical reinforcement of epithelial structure. Beyond direct transcriptional regulation, tryptophan metabolites can also indirectly maintain tight junction integrity by alleviating oxidative stress and promoting DNA repair. In intestinal injury caused by inflammation or radiation, excessive reactive oxygen species (ROS) disrupt tight junction proteins. Recent studies have found that IPA can alleviate oxidative stress and neuroinflammation by activating the Wnt1/STAT3 signaling pathway (56); meanwhile, in intestinal epithelial cell models, indole-3-acetic acid (IAA) derived from Lactobacillus salivarius can trigger AHR-PARP1 axis-mediated DNA repair networks (57).

In summary, this synergistic action of “transcriptional regulation and cytoprotection” collectively constructs a critical defense line against pathogenic bacterial invasion and blocks the pathogenesis of necrotizing enterocolitis (NEC).

### 4.3 Chemical barrier: antimicrobial defense mediated by AhR-IL-22-STAT3 axis

The chemical barrier is primarily composed of antimicrobial peptides (AMPs) secreted by Paneth cells and mucins secreted by goblet cells, forming a sterile isolation zone covering the epithelial surface. Preterm infants have insufficient Paneth cell and goblet cell numbers or immature function, resulting in thinning of the intestinal mucus layer and deficient antimicrobial peptide secretion, making NEC-associated pathogenic bacteria more likely to contact and adhere to epithelial cell surfaces. As “chemical messengers,” tryptophan metabolites can powerfully compensate for this developmental defect by activating the AhR-IL-22-STAT3 cascade signaling pathway (58). Mechanistic studies indicate that the aryl hydrocarbon receptor can promote interleukin-22 secretion from type 3 innate lymphoid cells (59). IL-22 binds to epithelial cell receptors to activate STAT3, powerfully inducing accelerated expression and secretion of multiple defense factors by immature epithelial cells, including regenerating islet-derived proteins (Reg3β, Reg3γ) with broad-spectrum antimicrobial activity, α-defensins that disrupt bacterial cell membranes, and mucin 2 (MUC2) constituting the core of the intestinal mucus barrier (59, 60). In preclinical NEC interventions, microbiota-derived indole metabolites amplify host innate defense responses precisely through this pathway, constructing an antimicrobial barrier on the preterm infant intestinal lumen surface that blocks pathogenic bacterial colonization, thereby effectively reducing NEC incidence and severity.

### 4.4 Immune barrier: inhibition of NF-κB pathway and promotion of Treg differentiation

The core pathogenesis of NEC involves excessive activation of the intestinal immune system, with extremely active TLR4 signaling driving NF-κB to trigger catastrophic inflammatory storms, while the preterm infant intestine is severely deficient in regulatory T cells (Tregs) capable of suppressing inflammation. Studies indicate that tryptophan metabolites can reinforce the intestinal immune barrier by inhibiting the NF-κB pathway and promoting regulatory T cell differentiation (61). NF-κB is a key transcription factor in inflammatory responses, and its activation induces the release of pro-inflammatory cytokines, including tumor necrosis factor-α, interleukin-6, and interleukin-8. Studies demonstrate that indole-3-propionic acid and indole-3-lactic acid (ILA) inhibit NF-κB signaling through dual mechanisms after activating AhR: on the one hand, inducing expression of suppressor of cytokine signaling 2 (SOCS2) to promote degradation of pro-inflammatory proteins (62); on the other hand, activated AhR may undergo physical interaction (cross-talk) with the RelA/p65 subunit of NF-κB, spatially blocking NF-κB binding to its target gene promoters (62, 63).

**Figure 2:**
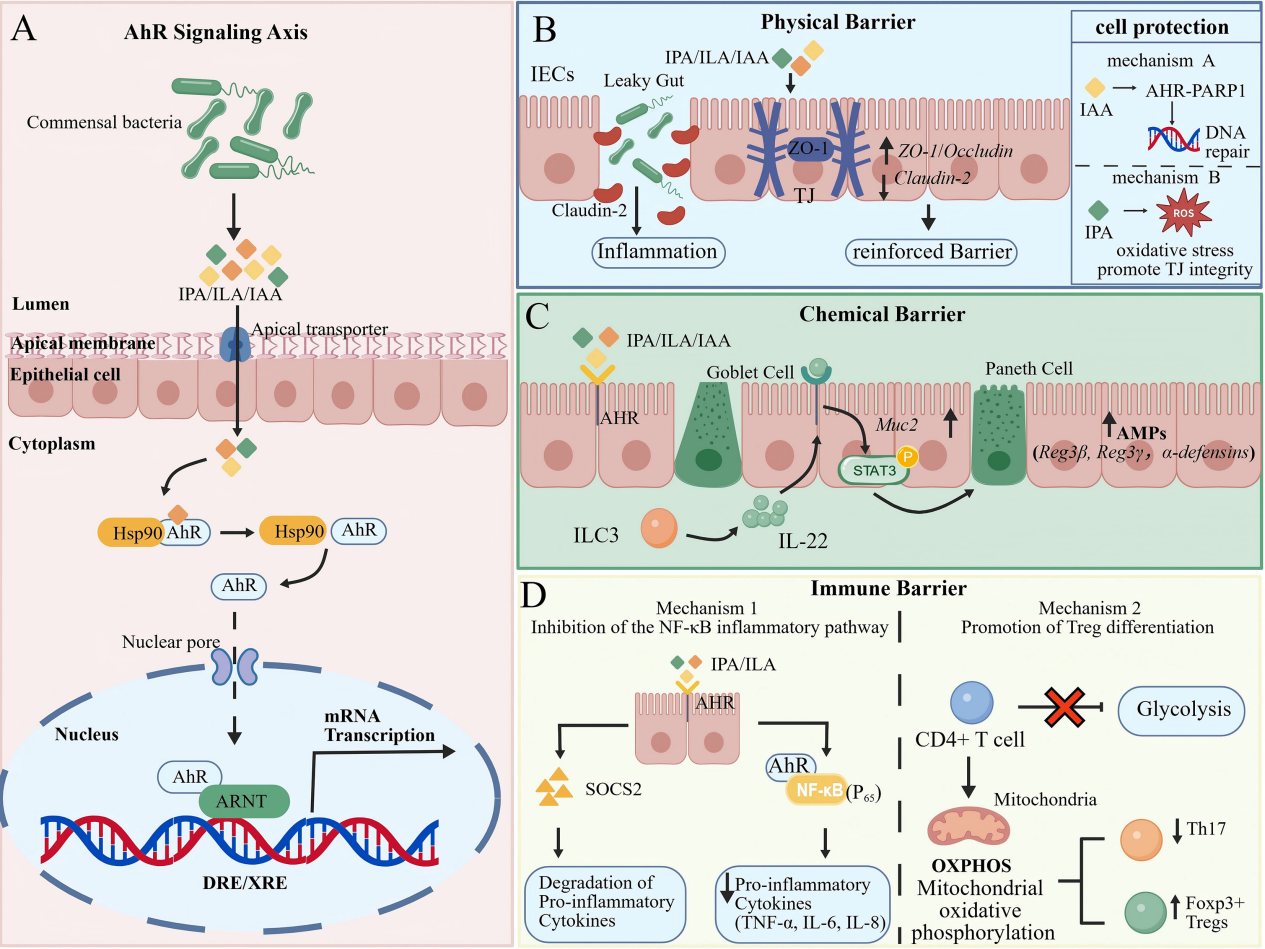
Tryptophan metabolites reinforce the multi-layered intestinal barrier via the AhR signaling axis. **(A)** The AhR Signaling Hub: Commensal bacteria-derived indole metabolites, such as indole-3-propionic acid (IPA), indole-3-lactic acid (ILA), and indole-3-acetic acid (IAA), serve as ligands for the aryl hydrocarbon receptor (AhR). Upon ligand binding in the cytoplasm, AhR dissociates from its chaperone protein Hsp90, translocates into the nucleus, and forms a heterodimer with the AhR nuclear translocator (ARNT). This complex binds to dioxin/xenobiotic response elements (DRE/XRE) to initiate the transcription of barrier-protective genes. **(B)** Physical Barrier Enhancement: AhR activation strengthens epithelial tight junctions by transcriptionally upregulating ZO-1 and Occludin while downregulating the pore-forming protein Claudin-2. Furthermore, IAA promotes DNA repair through the PARP1 axis, and IPA maintains junctional integrity by neutralizing reactive oxygen species (ROS). **(C)** Chemical Barrier Defense: Tryptophan-derived indoles stimulate type 3 innate lymphoid cells (ILC3s) to produce IL-22, which subsequently activates STAT3 signaling in intestinal epithelial cells. This cascade triggers Mucin 2 secretion from Goblet cells and the release of antimicrobial peptides (AMPs) from Paneth cells, thereby enhancing the mucosal isolation zone. **(D)** Immune Barrier Homeostasis: AhR signaling suppresses excessive inflammation through two distinct mechanisms: (1) inducing SOCS2-mediated degradation of pro-inflammatory proteins and interfering with NF-κB (p65) transcriptional activity via physical cross-talk; and (2) driving a metabolic shift from glycolysis to oxidative phosphorylation (OXPHOS) in CD4^+^T cells, promoting Foxp3^+^ regulatory T cell (Treg) differentiation and restoring the Treg/Th17 balance. Created with BioGDP.com (50).

Additionally, cellular function is highly dependent on metabolic patterns. Studies indicate that IPA can specifically drive CD4^+^ T cell metabolic remodeling from glycolysis toward mitochondrial oxidative phosphorylation (OXPHOS) (44). This metabolic reprogramming is precisely the key to inducing differentiation of immature T cells toward Foxp3^+^ Tregs while inhibiting pro-inflammatory Th17 cell differentiation. By replenishing tryptophan metabolites, local Treg/Th17 immune imbalance in the intestines of NEC patients can be effectively corrected, preventing inflammatory response-mediated autophagic necrosis of intestinal tissue (**Figure 2**).

In summary, the barrier-reinforcing effect of tryptophan metabolites on the intestinal barrier is not an isolated event but rather a systematic protective mechanism highly aligned with core pathological processes of necrotizing enterocolitis (NEC). In the preterm infant population, delayed colonization or insufficient abundance of specific commensal bacterial populations, such as Bifidobacterium and Lactobacillus, leads to insufficient generation of barrier-protective indole derivatives, thereby exposing the immature intestine to NEC pathogenic risks. Therefore, in future research and clinical translation, targeted supplementation of tryptophan metabolites with defined aryl hydrocarbon receptor (AhR) affinity, or screening of probiotic strains with stable indole-producing capacity, to synergistically repair physical barrier defects, enhance chemical defense functions, and inhibit excessive inflammatory responses, holds promise for development as a multi-target, mechanism-oriented precision prevention and treatment strategy for NEC.

## 5 Multi-level research evidence

Extensive preclinical studies and clinical observations provide multi-dimensional evidence supporting tryptophan metabolites in reinforcing the intestinal barrier to prevent necrotizing enterocolitis. Gut dysbiosis has been widely recognized as a core driver of NEC pathogenesis (10, 64), with its mediated metabolite dysregulation and TLR4 inflammatory cascades representing current research hotspots in this field (65, 66). In recent years, researchers have not only revealed novel targets such as L-cysteine metabolites regulating ferroptosis through the IL-6/STAT3 pathway (67), but have also increasingly focused on tryptophan metabolites. The following section systematically reviews the latest evidence of tryptophan metabolite-mediated intestinal protection from three dimensions: animal models, organoid *in vitro* culture, and clinical cohorts.

### 5.1 Animal model studies

Animal model studies provide direct evidence for tryptophan metabolites protecting the intestinal barrier. Research demonstrates that in mouse models of necrotizing enterocolitis, supplementation with tryptophan or direct administration of indole-3-propionic acid and indole-3-lactic acid can significantly reduce intestinal tissue injury scores and improve survival rates (45, 68). Notably, maternal supplementation with gut-derived tryptophan metabolite indole-3-propionic acid during pregnancy can alleviate colitis susceptibility in offspring of maternal immune activation models (45), suggesting that this metabolite has transplacental protective effects. Additionally, the colonization protective efficacy of probiotics is highly correlated with their ability to produce indole metabolites. Studies indicate that Bifidobacterium breve M-16V and Bifidobacterium infantis YLGB-1496 can effectively prevent neonatal NEC and food allergy by enriching microbiota-derived IPA and activating host aryl hydrocarbon receptor (AhR) signaling axis (69, 70); Lactiplantibacillus plantarum JY039 has been confirmed to improve necrotizing enterocolitis through intestinal stem cell regeneration (71). These studies collectively demonstrate that specific indole-producing strains and their metabolites can resist NEC occurrence through dual mechanisms of inhibiting inflammation and promoting epithelial repair.

### 5.2 Organoid model studies

Human intestinal organoid studies provide compelling evidence that tryptophan metabolites directly promote epithelial cell proliferation and differentiation. Intestinal organoids are three-dimensional culture systems derived from intestinal stem cells capable of simulating intestinal epithelial physiological functions. In recent years, multiple studies based on murine and human organoids have demonstrated that tryptophan metabolites (such as I3A, indole, etc.) can bypass complex *in vivo* microenvironments to directly act on Lgr5^+^ intestinal stem cells at crypt bases, promoting their proliferation and differentiation. The tryptophan metabolite I3A can powerfully drive Lgr5^+^ stem cell proliferation by activating AhR to link morphogenetic signaling pathways such as Wnt (72), thereby accelerating damaged mucosal regeneration and restoration of barrier integrity at the physical level; indole can significantly induce stem cell differentiation toward specific secretory cell lineages (enteroendocrine cells) through AhR-dependent pathways (73); Cetobacterium somerae ZNN-1 promotes goblet cell differentiation through glutamine-mediated Notch signaling inhibition (74), providing a mechanistic basis for probiotic promotion of intestinal barrier repair. This pro-regenerative capability directly targeting the stem cell niche enables tryptophan metabolites to accelerate recovery of damaged intestinal barriers, preventing bacterial translocation and systemic infection. Organoid studies provide direct cell biological evidence for mechanisms by which tryptophan metabolites reinforce the intestinal barrier.

### 5.3 Clinical cohort observations

Multi-omics analyses of clinical cohorts further corroborate preclinical findings, revealing strong correlations between metabolite deficiency and NEC pathogenesis risk. Metabolomic analyses demonstrate that compared to fecal and urine samples from NEC patients and healthy preterm infants, indole substances are significantly reduced in NEC patients (12). The root cause of this metabolic deficiency lies in severe gut dysbiosis—Proteobacteria are abnormally enriched in NEC patient intestines, while indole-producing Firmicutes and Bifidobacterium are significantly depleted (24, 75). Longitudinal analyses indicate that intestinal metabolite succession in extremely preterm infants is profoundly influenced by delivery mode and early feeding strategies (12, 76), providing new tools for early NEC prediction.

Clinical intervention studies also confirm that continuous supplementation with specific multi-strain probiotics (such as Bifidobacterium longum subsp. infantis) or enrichment of butyrate/indole-producing bacteria can significantly optimize intestinal bacterial metabolite patterns, thereby reducing risks of NEC and late-onset sepsis in very low birth weight infants (77–79).

Clinical intervention studies also confirm that bacterial metabolite patterns in infants receiving multi-strain probiotics correlate with late-onset sepsis risk (80). The latest clinical randomized controlled trials demonstrate that continuous supplementation with Bifidobacterium longum subsp. Infantis can reduce NEC risk in very low birth weight infants (81), providing clinical evidence for probiotic prevention of NEC through metabolic function remodeling. Additionally, in very low birth weight infants without culture-confirmed sepsis or NEC, the presence of butyrate-producing bacteria correlates with favorable clinical outcomes (82), further supporting the importance of beneficial bacterial metabolites in preterm infant health. These clinical cohort studies confirm that indole substances are significantly reduced in NEC patients, and the lack of indole-producing bacteria strongly correlates with NEC pathogenesis, providing clinical evidence for tryptophan metabolites as NEC prevention strategies.

Despite multi-dimensional existing evidence supporting the protective effects of tryptophan metabolites, their clinical translation in the extremely preterm infant population still faces challenges. Organoid studies predominantly derive from adult or term infant intestinal tissues, with metabolic characteristics and barrier functions significantly different from those of preterm infants. Although clinical cohort studies confirm reduced indole substances in NEC patients, they lack stratified analysis of key factors such as gestational age, birth weight, and feeding mode, making it difficult to determine whether tryptophan metabolite reduction is a cause or consequence of NEC (**Table 1**). Therefore, future research needs to establish preterm infant-specific animal models and organoid models and conduct prospective cohort studies to more accurately evaluate the role of tryptophan metabolites in preterm infant NEC prevention.

**Table 1:** Summary of tryptophan metabolites and microbiota in different research models.

Research dimension	Key models/subjects	Key metabolites/strains	Core biological effects & mechanisms	Representative evidence
Animal Models	Mouse NEC model; Maternal immune activation model	IPA, ILA; Bifidobacterium adolescentis, B. infantis	Reduces tissue damage and improves survival;Exerts transplacental protection, reducing offspring susceptibility;Activates AhR signaling; inhibits TLR4-mediated inflammation.	(45, 68–71)
Organoid Models	Mouse/human intestinal organoids (3D)	I3A, Indole; Cetobacterium somerae	Acts directly on Lgr5^+^ intestinal stem cells to promote proliferation; Accelerates mucosal regeneration via AhR-Wnt/Notch pathways; Induces goblet/enteroendocrine cell differentiation; repairs chemical barrier.	(72–74)
Clinical Cohorts	Preterm infants; VLBW infants	Indole derivatives; Bifidobacterium spp., Lactobacillus spp.	Metabolic deficiency: Indole levels are significantly reduced in NEC patients; Microbial succession: Metabolite profiles influenced by delivery mode and feeding; Intervention efficacy: Probiotics optimize metabolic patterns and reduce NEC incidence.	(12, 24, 75–82)

## 6 Therapeutic potential and future directions

Tryptophan metabolites demonstrate tremendous potential from early diagnosis to targeted therapy in preventing necrotizing enterocolitis (NEC) in preterm infants. However, translation from basic research to clinical application requires overcoming preterm infant-specific physiological barriers and delivery route challenges. This chapter will systematically elaborate intervention strategies based on tryptophan metabolism, constraints of preterm infant physiological specificity on clinical application, current research limitations, and future development directions.

### 6.1 Early diagnosis and intervention strategies based on tryptophan metabolism

#### 6.1.1 Early diagnosis: metabolites as NEC risk biomarkers

Dynamic monitoring of aryl hydrocarbon receptor ligand levels in neonatal feces holds promise as a non-invasive biomarker for early identification of high-risk NEC infants. Longitudinal cohort studies demonstrate that the dynamic evolution of fecal tryptophan metabolite profiles in extremely preterm infants closely correlates with NEC pathogenesis risk, with relative abundances of metabolic pathways significantly influenced by delivery mode and feeding type (12). This finding provides a theoretical basis for establishing NEC early warning systems based on metabolite profiles.

#### 6.1.2 Probiotic intervention: repairing endogenous metabolic flux

Probiotic intervention directly increases intestinal tryptophan metabolite levels by supplementing strains capable of producing indole derivatives, such as Bifidobacterium infantis and Lactobacillus plantarum (70). The latest meta-analyses confirm that probiotic supplementation can significantly reduce NEC, sepsis, and mortality in preterm infants and very low birth weight infants (83, 84), providing the highest level of evidence-based medical evidence for probiotic NEC prevention. However, limitations of probiotic strategies cannot be overlooked: their efficacy highly depends on colonization capacity and metabolic activity of strains in preterm infant intestines, and there is potential risk of opportunistic sepsis in infants with extremely low immune function (85, 86). Therefore, probiotic interventions require the establishment of precise screening criteria based on strain safety grading and host immune status assessment.

#### 6.1.3 Prebiotic intervention: shaping metabolically supportive microenvironments

Prebiotic interventions such as human milk oligosaccharides (HMOs) indirectly elevate intestinal indole metabolite levels by selectively promoting proliferation and metabolic activity of endogenous indole-producing bacteria such as infant-type Bifidobacterium and Lactobacillus (87, 88). HMOs naturally present in human milk and AhR ligands (such as IAA, indole-3-sulfate) form dual inputs, jointly reinforcing the preterm infant intestinal barrier (87, 89). The advantage of prebiotic interventions lies in their physiological compatibility and high safety profile, but their effects depend on whether preterm infant intestines already harbor target bacterial populations capable of being selectively promoted. For extremely preterm infants with severe microbial deficiency, the onset window may be delayed (90), suggesting that intervention timing should be selected based on microbial colonization status.

#### 6.1.4 Postbiotic intervention: direct supplementation of downstream indole metabolites

Postbiotic interventions directly supplementing tryptophan metabolites such as indole-3-propionic acid or indole-3-lactic acid represent new trends in microbiota-targeted therapy. Compared to live bacterial preparations, postbiotics avoid risks of bacteremia in preterm infants with low immunity, offering higher safety and dose controllability (91, 92). The latest preclinical multi-omics studies have confirmed that exogenous supplementation of purified IPA and ILA monomers demonstrates pro-healing effects in multiple colitis animal models, effectively blocking bacterial translocation and significantly improving host survival rates (54, 93).

### 6.2 Preterm infant physiological specificity and precision medicine considerations

Unique physiological characteristics of preterm infants pose special safety requirements for clinical application of tryptophan metabolites. First, preterm infants have immature liver and kidney function with low tryptophan metabolic enzyme activity, potentially leading to metabolite accumulation and increased toxicity risk (12). Second, preterm infants have high blood-brain barrier permeability, and excessive tryptophan metabolized through the kynurenine pathway may generate neurotoxic quinolinic acid, thereby damaging the central nervous system (27). Additionally, preterm infants have weak intestinal absorption capacity, and oral bioavailability of tryptophan metabolites may be significantly lower than in term infants (12). Therefore, determining preterm infant-specific safe dose ranges is crucial.

Therefore, future clinical applications must rely on precision medicine strategies: (1) identifying high-risk infants with metabolite deficiency through metabolomic detection of baseline indole substance levels in preterm infant feces (12); (2) formulating individualized intervention protocols based on gut microbiota composition and tryptophan metabolic enzyme gene polymorphisms (94); (3) improving tryptophan metabolite bioavailability using human milk fortifiers or formula additives (12); and (4) establishing dynamic monitoring systems to adjust intervention doses based on infant intestinal barrier function indicators (such as intestinal permeability, inflammatory cytokine levels) (75).

### 6.3 Research limitations

Although tryptophan metabolites demonstrate broad prospects in NEC prevention, current research still has core limitations. First, existing mechanistic evidence predominantly derives from animal models or adult studies, with limited direct evidence specific to preterm infants, and specific direct intervention data targeting the preterm infant population are extremely scarce (12, 95). Concurrently, different independent studies exhibit significant clinical heterogeneity in metabolite dosing, administration routes, and frequency, making precise data integration and effect size estimation difficult. Additionally, multiple indole derivative molecules (such as IPA, ILA, IAA, IAld, etc.) coexist within the complex intestinal microenvironment, and their competitive or synergistic mechanisms at the aryl hydrocarbon receptor (AhR) level remain unclear (96). Furthermore, AhR signaling exhibits high ligand dependence and double-edged sword effects: microbiota-derived indole metabolites as physiological ligands can effectively reinforce the barrier, while persistent supraphysiological activation by environmental toxins (such as dioxins) disrupts barrier integrity (97). Coupled with a lack of long-term prospective follow-up, the impact of such targeted interventions on long-term neurodevelopment, immune programming, and metabolic syndrome risk in preterm infants remains to be clarified (79). Therefore, precisely defining the safe therapeutic window and optimal ligand combinations for tryptophan metabolites will be the core challenge for future progression toward pediatric clinical trials.

### 6.4 Future research directions

Based on the aforementioned limitations, future research in the field of tryptophan metabolites and NEC prevention should focus on multi-dimensional frontiers of translation. First, during early clinical translation, rigorous Phase I/II dose-escalation and safety trials targeting high-risk preterm infants are urgently needed to establish optimal pharmacokinetic models based on gestational age and birth weight. Subsequently, artificial intelligence can be leveraged to deeply integrate clinical and multi-omics features, progressively constructing individualized NEC precision intervention and early warning systems (98). Second, at the mechanism exploration level, frontier technologies such as single-cell sequencing and spatial transcriptomics should be utilized to systematically resolve competitive binding or synergistic amplification effects among multiple metabolic molecules at the AhR level within complex intestinal microenvironments, and deeply investigate their molecular mechanisms in non-canonical pathways such as anti-ferroptosis and DNA repair as well as synergistic enhancement with human milk/prebiotics (57, 99). Additionally, regarding the “double-edged sword” effect of AhR signaling, future drug development should not blindly pursue potent synthetic agonists but rather commit to optimizing targeted delivery systems for specific tryptophan metabolites or exploring optimal postbiotic combinations while simultaneously evaluating potential perturbation of NICU environmental exposures (such as additive Tween 80) on intestinal tryptophan metabolic pathways (100). Finally, long-term follow-up cohorts for extremely preterm infants must be established to systematically confirm safety regarding long-term outcomes such as prevention of quinolinic acid neurotoxicity, thereby providing lifelong health protection for preterm infants (101).

## 7 Conclusion

Tryptophan metabolites play a core role in preventing necrotizing enterocolitis in preterm infants by reinforcing the intestinal barrier through the aryl hydrocarbon receptor axis. As high-affinity ligands for the aryl hydrocarbon receptor, microbiota-derived indole derivatives comprehensively enhance intestinal physical, chemical, and immune barrier functions through multiple mechanisms, including upregulating tight junction protein expression, promoting antimicrobial peptide and mucin secretion, inhibiting the NF-κB pathway, and promoting regulatory T cell differentiation.

However, the unique developmental and intensive care environment of preterm infants disrupts this physiological homeostasis. Due to delayed gut microbiota colonization and reduced diversity, tryptophan metabolism in preterm infants exhibits abnormal bias, leading to significantly reduced production of protective indole derivatives. This deficiency in core metabolic signals directly causes insufficient AhR activation, rendering the already fragile intestine highly susceptible to a vicious cycle of barrier collapse and uncontrolled inflammation. Therefore, precisely reconstructing the “microbiota-metabolite” interaction in the preterm infant intestine becomes a key translational target for preventing NEC pathogenesis.

Clinically, ecological modulation strategies including human milk feeding, specific probiotic supplementation, and prebiotic interventions can effectively promote beneficial bacterial colonization and restore physiological abundance of endogenous indole derivatives, demonstrating tremendous preventive potential. Future work still requires more rigorous basic research and prospective clinical trials to further verify the efficacy and long-term safety of targeted tryptophan metabolite interventions in NEC prevention, thereby advancing clinical translation application of this frontier strategy.
